# Effectiveness of acceptance and commitment therapy for suicidality: a systematic review and meta-analysis

**DOI:** 10.1017/neu.2025.18

**Published:** 2025-04-17

**Authors:** Ju Hyun Tak, Seo-Eun Cho, Seong-Jin Cho, Seung-Gul Kang, Seung Min Bae, Kyoung-Sae Na

**Affiliations:** 1 Department of Psychiatry, Gil Medical Center, Incheon, Republic of Korea; 2 Department of Psychiatry, Gachon University College of Medicine, Gil Medical Center, Incheon, Republic of Korea

**Keywords:** Acceptance and commitment therapy, suicidality, suicidal ideation, systematic review, meta-analysis

## Abstract

Acceptance and commitment therapy (ACT) is recognised as an effective treatment for a variety of mental illnesses. Several meta-analyses have reported the efficacy of ACT in various mental and physical conditions, including depression, anxiety, and pain, but not for suicidality. This study aimed to determine the therapeutic effectiveness of ACT on suicidality through a systematic review and meta-analysis. Electronic databases such as PubMed, Embase, Scopus, and the Cochrane Library were searched for studies. The primary outcome measure was the effectiveness of ACT for suicidality which includes suicidal ideations and attempts. This systematic review and meta-analysis included eight studies, all of which were judged to have a high risk of bias. In the meta-analysis, the pooled standardised mean difference for suicidal ideations was 1.122 (95% confidence interval (CI) = 0.261 to 1.982). This meta-analysis suggests that ACT is effective for reducing suicidal ideation, but the high risk of bias across studies should be considered as a major limitation. Further well-designed studies are needed to confirm these findings.

Significant outcomes

1. ACT demonstrated promising efficacy in reducing suicidal ideation, with a significant effect size (SMD = 1.122, 95% CI = 0.261 to 1.982) across the included randomised trials.

Limitations

1. All included studies exhibited a high risk of bias.
2. Only a limited number of studies were included in the study.
3. Most, studies were conducted over relatively short durations.
4. The included studies were geographically concentrated, which may limit the generalizability of the findings to broader populations.

## Introduction

Suicide is a tragic outcome of mental health distress that remains a leading cause of death worldwide, with over 703,000 mortalities annually (World Health Organization, 2021). This highlights the urgent need for effective interventions to prevent and manage suicidal ideation and suicide attempts. Unfortunately, there is no simple treatment that can prevent suicide. Regarding pharmacologic options, clozapine for schizophrenia (Masdrakis & Baldwin, 2023), esketamine for major depressive disorder (Zhdanava *et al*., 2023), and lithium for bipolar disorder (Smith & Cipriani, 2017) are effective in preventing suicide, but these cases account for only a small percentage of all suicide deaths.

A recent umbrella review reported the effectiveness of cognitive-behavioural therapy (CBT) for reducing suicidal ideation and attempts (Wu *et al*., 2022). Dialectical behaviour therapy (DBT) was effective in reducing suicide attempts but had no significant effect on suicidal ideation (DeCou *et al*., 2019), while mentalization-based therapy was not effective in reducing self-harm compared with controls (Hajek Gross *et al*., 2024). Meanwhile, there is a lack of systematic reviews and meta-analyses regarding the effectiveness of major psychotherapeutic approaches, including acceptance and commitment therapy (ACT), in reducing suicidality.

**Figure 1.**
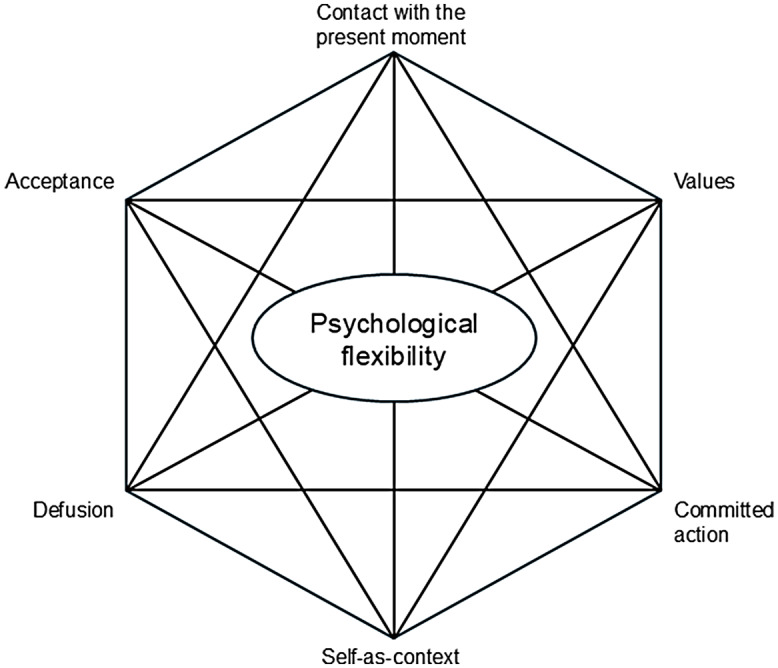
The hexaflex model of Acceptance and Commitment Therapy.

ACT is considered one of the ‘third-wave’ behavioural therapies, which emphasises acceptance and nonjudgmental attitudes toward thoughts and feelings (Hayes *et al*., 2012). ACT is action-oriented and focuses on helping people commit to values-based goals and move forward in their lives despite the presence of painful inner experiences. ACT is based on the hexaflex model (Figure 1), which visually represents six core therapeutic processes: acceptance, defusion, self-as-context, contact with the present moment, value clarification, and committed action (Belisle & Dixon, 2022). The term ‘hexaflex’ derives from the Greek word ‘hexa’ meaning six and ‘flex’ meaning flexibility, reflecting ACT’s core concept of promoting psychological flexibility through six core therapeutic processes. These interconnected processes aim to promote psychological flexibility, which refers to staying in contact with the present moment while remaining open to a wide range of thoughts, feelings, and bodily sensations and making choices based on personal values and the current situation. The six factors of the hexaflex can be briefly explained as follows. Acceptance is the act of fully and wholeheartedly embracing reality as it is. This is not a passive or resigned attitude but rather an active and intentional process. For instance, if someone were unjustly imprisoned and practiced acceptance, they would not spend their time helplessly blaming their misfortune or the world. Instead, they might actively work to raise awareness of their injustice, pursue an academic degree, learn a foreign language, or engage in fitness activities while in prison. In essence, acceptance involves willingly embracing one’s reality to create a foundation for doing the best one can within those circumstances. Defusion is a technique aimed at minimising the harmful influence of language. One of the most distinctive aspects of ACT compared to other third-wave cognitive-behavioural therapies is its strong foundation in Relational Frame Theory (RFT). RFT, in simple terms, posits that the human mind operates through a language-based system that shapes and governs our mental world. For example, the word lemon as a linguistic stimulus may evoke past experiences related to lemons. Upon seeing the word, a person might recall the sour taste of a lemon and even experience a physiological response, such as increased salivation. Defusion works to reduce this linguistic influence. If the person were to rapidly repeat the word lemon dozens of times, they would eventually stop associating it with sensory or physiological responses. Instead, they would perceive the word merely as a string of letters without attaching the meaning of sourness or salivation to it. Defusion is particularly effective at diminishing the linguistic influences that contribute to rumination and worry, helping individuals detach from unhelpful thought patterns. Contact with the present moment refers to being fully present in the here and now, a concept closely aligned with mindfulness. Often, while we may be physically awake, our minds are occupied with ruminating on past events or worrying about the future. Contact with the present moment, like mindfulness, helps increase our connection to the present, enabling us to focus on and engage with the current moment more effectively. Self-as-context is the ability to see oneself not as a fixed entity but as a constantly evolving and dynamic being that changes with context and time. For example, if someone holds a rigid self-concept like, ‘I never make mistakes; I’m always perfect’, they might become deeply disappointed in themselves and find it hard to accept even minor errors. However, a person with a strong sense of self-as-context might say, ‘In such a busy and challenging situation, it’s natural to make mistakes’, allowing for self-understanding and self-compassion. Values serve as a compass for life, guiding our actions and decisions. They represent aspirations we strive toward but can never fully achieve. For instance, if someone holds the value of ‘becoming a better person’, achievements like earning a certification or getting a promotion may serve as intermediate goals but cannot represent the value itself. Finally, committed action refers to taking concrete steps aligned with one’s values. Values-based committed action is particularly crucial for individuals with depression, as it helps them overcome inertia and lack of motivation, fostering recovery.

The hexaflex model is not linear but circular, as depicted in Figure 1. Therapists can start working at any point within the hexaflex depending on the client’s state and move freely between factors as the process unfolds. It is akin to the therapist and client forming a pair and navigating through the hexaflex factors, stepping together in a kind of ‘ACT dance’.

While a growing body of research supports the efficacy of ACT for various mental health concerns, such as depression, anxiety, and chronic pain (Gloster *et al*., 2020), its usefulness for suicidality warrants further investigation. Only one systematic review has reported on the effectiveness of ACT for suicidality, which included two case reports, two before-and-after comparisons, and one randomised controlled trial (RCT) (Tighe *et al*., 2018). In the case reports by Luoma & Villatte (2012), ACT was applied to two participants, both of whom showed significant improvement in suicidal ideation (Luoma & Villatte, 2012). Similarly, Razzaque (2013) reported case studies involving three participants who underwent one-on-one ACT sessions for 2–3 weeks (Razzaque, 2013). Among them, one individual experienced a significant reduction in self-harm and suicidal ideation, while the other two showed a decrease in expressions of self-harm or suicidal thoughts. Ducasse *et al*. (2014) recruited 37 psychiatric patients without a control group (Ducasse *et al*., 2014). The researchers implemented ACT in weekly 2-hour sessions over a total of 7 weeks. They reported that suicidal ideation measured at 1- and 3-month post-intervention significantly decreased. Walser *et al*. (2015) conducted a study involving 981 veterans, providing a total of 12–16 sessions of depression-focused ACT without a control group. (Walser *et al*., 2015). They reported a 20.5% overall reduction in the prevalence of suicidal ideation among the participants. Tighe *et al*. (2017) implemented a mobile ACT app for six weeks among youth from Australia’s First Nations people. (Tighe *et al.,* 2017). The study included a wait-list control group to compare with the intervention group. The results showed that the intervention group experienced a 42% reduction in subjective depression, as measured by the Patient Health Questionnaire-9, which was statistically significant compared to the control group (*p* = 0.02). Although suicidal ideation in the intervention group decreased by 30%, the difference compared with the control group was not statistically significant (*p* = 0.3). Based on those studies, the authors concluded that the lack of evidence makes it difficult to determine the effectiveness of ACT in reducing suicidal ideation. As evidenced by the characteristics of the individual studies included in this systematic review, there was only one RCT, and even in that study, the effect of ACT on suicidal ideation showed no statistically significant difference compared to the control group. However, given that eight years have passed since this systematic review was published, we felt it was necessary to reassess the effectiveness of ACT on suicidality.

The present systematic review and meta-analysis aims to compile the research on ACT for suicidality and determine if it effectively reduces this. We hypothesised that ACT is effective in reducing suicidality because it encourages psychological flexibility to tolerate pain and take actions that move toward meaning and value in life.

## Material and methods

**Figure 2.**
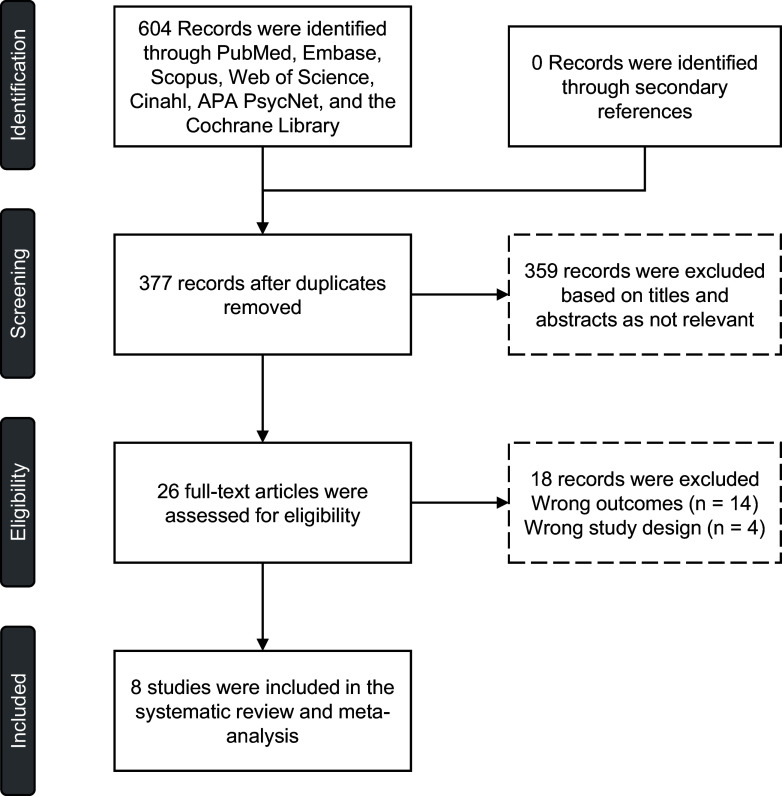
The PRISMA flow diagram.

We conducted this study following the PRISMA guidelines (Figure 2).

### Selection criteria

Electronic databases such as PubMed, Embase, Scopus, and the Cochrane Library were searched for studies, including only RCTs with no restrictions on language and publication period. Other types of studies such as cross-sectional case-control studies, observational studies, and pre-post comparison studies without appropriate controls were excluded. We included studies in which suicidal ideation or suicide attempts were the primary focus. Only studies that used valid measures for assessing suicidal ideation (e.g., Beck Scale for Suicide Ideation [BSS], Columbia Suicide-Severity Rating Scale [C-SSRS]) were included in the analysis.

### Participants

There was no age limit for the participants. We included individuals with experiences of nonsuicidal self-injury or suicidality (i.e., people with suicidal ideations and/or a previous suicidal attempt). Nonsuicidal self-injury is defined as the deliberate, self-inflicted destruction of body tissue without suicidal intent and for purposes not socially sanctioned (Zetterqvist, 2015).

### Interventions

We included studies that utilised ACT as a stand-alone or primary intervention for suicidality. Studies that used novel therapies that mixed ACT with various modalities or ACT combined with a particular CBT (e.g., DBT, mindfulness-based cognitive therapy, etc.) were deemed ineligible, and thus, those studies were excluded. We did not limit the form (individual or group), delivery (in-person or virtual), duration, and frequency of the intervention.

### Outcomes

We included studies that measured suicidality with instruments that have well-established validity. The primary endpoint was the change in suicidality before and after intervention between the experimental and control groups. For studies with a follow-up period after ACT, we analysed the results at the last time period tracked.

### Search strategy

Two researchers independently searched PubMed, Embase, Scopus, and the Cochrane Library using the following search terms: suicid* AND Acceptance and Commitment Therapy. The search period was set from the database’s inception to January 31, 2024. Since there were no language restrictions, for non-English articles, we asked native speakers familiar with the language and culture to translate them.

After excluding duplicate articles, two researchers independently conducted the initial screening using only the title and abstract. Any disagreements were initially discussed between the two researchers then resolved by a third researcher if a consensus was not reached. Full texts were obtained for all screened articles and reviewed independently by two additional researchers. Any disagreements were resolved in a similar fashion.

### Data extraction

Two researchers independently extracted the following data: publication year, country, first author, number of subjects (i.e., the number and clinical characteristics of each control and experimental group), ACT details (i.e., form, number of sessions, and duration), mean outcome measures and standard deviations at baseline and endpoint, and follow-up details. Any differences in the extracted data between the two researchers were subject to peer review and discussions to reach a consensus. All data were managed and processed using Covidence.

### Risk of bias assessment

Quality assessment was conducted according to Risk of Bias 2.0 (RoB 2) (Boutron *et al*., Boutron *et al*., 2019). RoB 2 was first developed by the Cochrane Collaboration in 2008 to address issues such as low inter-rater reliability in some domains and difficulties in properly assessing the risk of bias. It underwent a revision in 2011. RoB 2 is thought to have addressed some of the issues raised in the previous version through changes such as assessing the risk of bias at the level of individual study results rather than outcomes, providing algorithms and signalling questions to guide risk of bias judgments for each domain, distinguishing between the effects of intervention assignment and adherence to intervention, and introducing an explicit process for evaluating the overall risk of bias. RoB 2 is divided into five categories: (1) bias arising from the randomisation process, (2) bias due to deviations from intended interventions, (3) bias due to missing outcome data, (4) bias in measurement of the outcome, and (5) bias in selection of the reported result. The overall bias risk level is estimated using the results of all five categories together.

### Statistical analysis

Comprehensive Meta-Analysis version 4 (Borenstein *et al*., 2022) was used for statistical analyses. The corresponding authors of the articles were contacted in case of any missing data. Given the use of different instruments for suicidality, we calculated the standardised mean differences (SMD) and 95% confidence interval (CI). A correlation coefficient of *r* = 0.5 was universally accepted and employed as a measure of the association between the pretreatment and posttreatment totals.(Wang *et al*., 2019). The random-effects model was applied; this assumes that the true effect size may vary between studies, beyond what is expected due to random error or sampling variability alone. However, the fixed effects model assumes that the true effect size is the same in all included studies and that any observed differences in effect sizes between studies are due to random error or sampling variability only (Riley *et al*., 2011).

If more than 10 individual studies were retrieved, we planned to perform subgroup analysis or meta-regression to identify variables associated with pooled statistical results (Deeks *et al*., 2019) and funnel plot test for assessing publication bias (Tarsilla, 2019). We decided to use Egger’s regression test as an alternative to the funnel plot to assess publication bias when the number of studies included in the meta-analysis is fewer than ten. Heterogeneity was defined using *I* ^*2*^ statistics, with *I^2^* values of 25, 50%, and 75% indicating low, moderate, and high levels of heterogeneity, respectively (Higgins *et al*., 2003).

## Results

### Study characteristics

The systematic review included eight studies, with their characteristics shown in Table 1. The country with the most studies conducted was Iran, with four studies (Sara *et al*., 2021; Bagheri-Sheykhangafshe *et al*., 2022; Shareh and Robati, 2022; Bahram *et al*., 2023), followed by France (Ducasse *et al*., 2018), the United States (Barnes *et al*., 2021), Egypt (El-Sayed *et al*., 2023), and Australia (Tighe *et al.,* 2017) with one study each. The most commonly utilised measure of suicidality was the BSS (Beck and Steer, 1991) (5 studies), followed by the C-SSRS (Posner *et al*., 2011) (2 studies) and the Depressive Symptom Inventory-Suicidality Subscale (DSI-SS) (Joiner *et al*., 2002) (1 study). Most studies were conducted over a timeframe of 1–3 months.

**Table 1.** Study characteristics

Study ID	Country	Participants	Outcome measure	Intervention group				Control group		
				Treatment	Treatment duration	Sample size	Mean age(SD)	Treatment	Sample size	Mean age (SD)
Bahram *et al.,* 2023	Iran	Male adolescents aged 13–18 in. Ardbil, surveyed in the second half of 2021	BSSI	ACT, eight 90-min sessions, weekly	8 weeks	15	–	No intervention	15	–
Barnes *et al.,* 2021	USA	Patients admitted to a VHA psychiatric inpatient unit at an urban VA medical centre because of the risk of suicide, per patients’ admission note	C-SSRS	ACT + TAU, ‘ACT for Life’	30 days or 90 days	35	42(22–70)	TAU	35	49 (27–73)
El-Sayed *et al.,* 2023	Egypt	Clients with a DSM-5 bipolar diagnosis	BSSI	ACT + TAU, eight 90-min sessions, weekly	8 weeks	30	33.47(6.91)	TAU + Waitlist	30	35.10 (7.81)
Tighe *et al.,* 2017	Australia	Indigenous Australians aged 18–35 years	DSI-SS	ACT, ibobbly programme	6 weeks	31	27.48(9.54)	Waitlist	30	24.97 (6.28)
Ducasse *et al.,* 2018	France	Patients from the Department of Psychiatric Emergency and Acute Care, Academic Hospital of Montpellier (France)	C-SSRS suicidal ideation subscore	ACT, seven 2-h sessions, weekly	7 weeks	21	38.34(12.73)	Progressive Relaxation Training	19	38.04 (11.08)
Sara *et al.,* 2021	Iran	Patients with Bipolar Disorder	BSSI	ACT, eight 90-min sessions, two per week	4 weeks	15	–	–	15	–
Shareh & Robati, 2022	Iran	All depressed conscripts referred to military counselling centres in the Khorasan Razavi Province of Iran	BSSI	ACT, eight 90-min sessions, weekly	8 weeks	30	–	Waitlist	30	–
Bagheri-Sheykhangafshe *et al.,* 2022	Iran	Students of Guilan University in 2021	BSTQ	ACT, eight 2-h sessions, weekly	8 weeks	15	–	No intervention	15	–

ACT, Acceptance and Commitment Therapy; BSSI, Beck Scale for Suicidal Ideation; BSTQ, Beck Suicide Thought Questionnaire; C-SSRS, Columbia Suicide-Severity Rating Scale; DSI-SS, Depressive Symptom Inventory-Suicidality Subscale; TAU, Treatment-As-Usual.

In Egger’s regression test, there was no significant publication bias (standard error [95% CI] = 2.86 [−3.19 to 21.4], *p* = 0.086). There seemed to be high heterogeneity (*I* ^*2*^ = 88.2) among the studies included.

### Efficacy of ACT

Only four of eight studies included in the systematic review were eligible for meta-analysis (Tighe *et al.,* 2017; Barnes *et al*., 2021; Bahram *et al*., 2023; El-Sayed *et al*., 2023). The four ineligible studies lacked at least one essential quantitative indicator necessary for conducting a meta-analysis. Despite multiple efforts to contact the corresponding and first authors of each study via email, we did not receive any responses. Consequently, these four studies were excluded from meta-analysis. Among the four eligible studies, the pooled SMD (95% CI) for suicidal ideation was 1.122 (95% CI = 0.261–1.982). With only four RCTs included in the meta-analysis, we were unable to perform the publication bias and meta-regression that was originally planned.

### Risk of bias

Overall, individual studies included in the systematic review had significant levels of bias (Table 2). All eight studies had ‘High’ in the Overall Risk Bias. Among subcategories, all eight studies had ‘High’ in the Measurement Outcome. The study by El-Sayed *et al*. (2023) had the lowest risk of bias, with four domains (Randomization Process, Intended Interventions, Missing Outcome Data, Selection of Reported Data) rated as ‘Low’. The study by Tighe *et al.*, (2017) followed, with three domains (Randomization Process, Intended Interventions, Missing Outcome Data) rated as ‘Low’. In contrast, none of the four studies conducted in Iran received a ‘Low’ rating in any domain

**Table 2.** Quality assessment of included studies using risk of bias 2.0 tool

Study	Randomization process	Deviation from intended interventions	Missing outcome data	Measurement of outcome	Selection of Reported Result	Overall Bias Risk
Bahram *et al.*, 2023	Some concerns	Some concerns	High	High	Some concerns	High
Barnes *et al.*, 2021	Low	Low	High	High	Low	High
El-Sayed *et al.*, 2023	Low	Low	Low	High	Low	High
Tighe *et al.*, 2017	Low	Low	Low	High	Some concerns	High
Ducasse *et al.*, 2018	Low	Some concerns	Low	High	Low	High
Sara *et al.*, 2021	Some concerns	Some concerns	High	High	Some concerns	High
Shareh & Robati, 2022	Some concerns	Some concerns	High	High	Some concerns	High
Farzin Bagheri-Sheykhangafshe, 2022	Some concerns	Some concerns	High	High	Some concerns	High

## Discussion

This systematic review and meta-analysis evaluated the effectiveness of ACT in reducing suicidality. The meta-analysis revealed a significant effect size across the 4 RCTs eligible for inclusion, suggesting that individuals undergoing ACT experienced a substantial reduction in suicidal ideation compared to controls. This outcome underscores the potential of ACT to address suicidal ideation effectively.

ACT conceptualises suicidality as a learned and reinforced response to emotional distress, rather than solely a symptom of mental illness (Chiles *et al*., 2019). This perspective allows for a more nuanced understanding of suicidal behaviour, recognising that it can occur in individuals with or without diagnosed mental disorders. ACT views suicidal behaviour as existing on a continuum of problem-solving methods, rather than as an absence of solutions. From the perspective of ACT, a suicidal crisis is fundamentally rooted in an individual’s relationship with their own distress. While suffering is an inevitable part of life, we are culturally conditioned to believe that we must eliminate or escape from uncomfortable thoughts, emotions, or sensations. This tendency to control, remove, or suppress negative thoughts, emotions, or sensations rather than accepting them is known as experiential avoidance. When individuals engaging in the experiential avoidance fail to control or eliminate their negative internal events, they may resort to increasingly avoidant strategies, with suicidal behaviour representing one such extreme. Researches consistently demonstrated that emotionally avoidant coping strategies are strongly associated with various psychopathological phenomena, including suicidal tendencies (Hayes *et al.,* 2012; Chiles *et al.,* 2019). In contrast to traditional emotion-focused problem-solving, which aims to resolve mental suffering, ACT’s values-based problem-solving approach treats mental suffering as a given and aims to help individuals respond to life’s challenges in accordance with their beliefs and values.

The key difference of ACT from other major CBT approaches lies in the RFT (Hayes *et al*., 2001). ACT directly targets the RFT and changes the context. For example, suicidal thoughts often arise from maladaptive relational networks (e.g., ‘I am worthless’ leading to ‘I should die’). ACT helps reframe these networks in a more adaptive way and changes the way we respond to certain stimuli based on their relational context. ACT can reduce the significance of suicidal ideas by altering the relational context of suicidal thoughts (i.e., seeing them as just thoughts rather than commands). Additionally, by fostering a nonjudgmental stance toward painful emotions, individuals can learn to manage distress without resorting to suicidal behaviours.

ACT also emphasises connecting with personal values and engaging in committed action aligned with those values. This focus on meaningful living can provide a sense of purpose and direction, potentially counteracting feelings of hopelessness often associated with suicidal ideation (Ramaci *et al*., 2019). Furthermore, the emphasis on mindfulness and awareness of the present moment can help individuals break cycles of rumination and catastrophic thinking that can fuel suicidal thoughts (Baer & Debra 2008, Ramaci *et al*., 2019). Clinicians can integrate ACT techniques into their therapeutic approach by practising mindfulness and acceptance strategies during and in between sessions. For example, they can guide patients in examining the thoughts that emerged during a suicidal crisis or use visualisation techniques, such as imagining placing distressing thoughts into clouds in a clear sky and allowing them to float by without judgment. Another effective method is to have patients complete values clarification exercises, helping them recognise their values across various domains and set specific behavioural goals aligned with those values (Hayes *et al.,* 2012; Chiles *et al.,* 2019).

Several limitations must be considered in our study. First, a high risk of bias was observed across all eight studies, with particular concerns regarding the randomisation process, deviations from intended interventions and selective reporting, especially in studies conducted in Iran. This high risk of bias raises concerns that the results of the meta-analysis would not be reliable. We believe the main reason for this issue is the lack of sufficient information available from individual studies. According to the algorithm of the RoB 2, if even one of the five individual items is classified as high risk, the overall risk of bias is also categorised as high risk. Notably, all studies were assessed as high risk in the ‘Measurement of Outcome’ domain due to insufficient information. As mentioned in Methods, we made several attempts to contact the corresponding authors of the studies included via email to obtain the missing information, but we did not receive any responses. These communication challenges likely led to a classification of high risk of bias. Second, the number of studies included in the analysis was limited; only eight out of 604 studies identified across multiple databases met the inclusion criteria for our systematic review. This may be attributed to the relative novelty of ACT compared with more established therapies such as CBT, although suicidality is infrequently studied even in these more established treatments. Ethical concerns inherent in suicide research also necessitate stringent safety protocols for participants at elevated risk (Lakeman & FitzGerald, 2009; Bhasin *et al*., 2022). Third, the preponderance of Iranian studies in this analysis is noteworthy, given the increasing suicide rates in the country. While the prevalence of suicide attempts in Iran remains below the global average, it has shown a significant upward trend, with suicide-related mortality rising from approximately 0.2 to 6.2 per 100,000 from 1991 to 2003 (Hossein Hassanian-Moghaddam & Zamani, 2017; Mahboobeh Asadiyun and Daliri, 2023). Additionally, recent data indicated a 60% increase in suicide rates between 2015 and 2019, thus underscoring the urgency for intervention(Hossein Hassanian-Moghaddam and Zamani, 2017; Mahboobeh Asadiyun and Daliri, 2023). This urgency likely drives research into various therapeutic approaches on suicidality, including ACT. Nevertheless, the geographical concentration of the included studies may limit the generalizability of our findings to populations outside Iran, potentially affecting the external validity of the meta-analysis results. Fourth, most studies included in this review had relatively short study periods. Most of the studies were conducted for 8 weeks or less, whereas only one study reported an outcome at 3 months (Barnes *et al*., 2021). This limits the ability of our manuscript to assess the long-term effectiveness of ACT on suicidality. There are significant challenges in conducting long-term studies on suicidality due to the rarity of events, resulting in low base rates and small samples (Lakeman and FitzGerald, 2009). This complexity makes it challenging to isolate the effects of a single therapeutic approach such as ACT in an RCT setting. Fifth, although the SMD indicates a considerable effect size, its interpretation requires significant caution. While no significant publication bias was detected, the potential influence of confounding factors (e.g., short study durations, small number of included studies) cannot be ruled out. Lastly, we were unable to perform a subgroup analysis or meta-regression to identify potential moderators of ACT (e.g., follow-up duration, intervention type, severity of suicidal ideation) due to the small number of studies included in the meta-analysis. This limitation may have hindered a more detailed and specific interpretation of our findings and, as a result, could make it challenging to directly apply our results in clinical practice.

Despite the limitations, our meta-analysis provides evidence supporting the efficacy of ACT for reducing suicidal ideation. By including 8 RCTs in the systematic review and 4 RCTs in the meta-analysis, we provided a higher level of evidence compared to the previous systematic review (Tighe *et al*., 2018), which included only a single RCT. This meta-analysis is the first quantitative assessment of the effect of ACT on suicidality, which was not possible in the previous review due to the heterogeneity of the studies included.

We suggest several aspects that future research should focus on. First, longitudinal studies with extended follow-up periods are needed. These would allow for a more comprehensive evaluation of the durability of treatment effects and provide stronger evidence for the sustained impact of ACT on suicidality and other psychiatric conditions. Second, studies with populations from diverse geographical regions and sociocultural settings are needed. Third, a structured or reproducible protocol of ACT would be necessary. ACT is a technique that emphasises psychological flexibility, and because of this, the developers did not initially create a structured protocol (Hayes *et al*., 1999). Therefore, ACT is often described with the term ‘transdiagnostic approach’. However, over time, as ACT has been optimised for use in various settings and conditions, cases of its application have increased, leading to the natural development of structured or optimised protocols (Bethay *et al*., 2022; Jeong *et al*., 2022). Currently, ACT is delivered through various formats, including one-on-one or group face-to-face sessions, pre-produced digital content, and interactive applications. Each of these formats has its own advantages and disadvantages. Having structured or reproducible protocols tailored to these representative settings would make it much easier to verify their effectiveness, and the results would also be more reliable. Fourth, researchers reporting RCTs should be encouraged to provide sufficient information to allow for the assessment of items required by credible risk of bias tools, such as RoB 2. Insufficient information can lead to an overall classification of high risk of bias, which may ultimately raise concerns about the reliability of meta-analysis results. Lastly, it is crucial to investigate the specific mechanisms of ACT that impact suicidality.

## Conclusions

This systematic review and meta-analysis suggested that suicidal ideation decreased among individuals who received ACT compared to controls. This outcome underscores the potential of ACT in effectively addressing suicidal ideation. However, due to the heterogeneity and the risk of bias issues, more well-designed RCTs with larger sample sizes are warranted. Future studies should aim to replicate these findings and to elucidate the mechanisms by which ACT reduces suicidality.
